# Comment on “Background Ionizing Radiation and the Risk of Childhood Cancer: A Census-Based Nationwide Cohort Study”

**DOI:** 10.1289/ehp.1509938

**Published:** 2015-08-01

**Authors:** Bobby R. Scott

**Affiliations:** Lovelace Respiratory Research Institute, Albuquerque, New Mexico, USA

Spycher et al. conducted a nationwide census-based cohort study to investigate whether the incidence of childhood cancer is associated with external exposure to natural background radiation from terrestrial gamma and cosmic rays. The authors claim their results suggest an increased risk of cancer among children exposed to external dose rates of background ionizing radiation of ≥ 200 nSv/h (1.8 mSv/yr) when compared with those exposed to < 100 nSv/h (0.9 mSv/yr). Furthermore, they claim the hazard ratios for each mSv increase in cumulative dose of external radiation are 1.028 (95% confidence interval [CI]: 1.008, 1.048) for any cancer, 1.036 (95% CI: 0.997, 1.077) for leukemia, 1.007 (95% CI: 0.964, 1.052) for lymphoma, and 1.042 (95% CI: 1.008, 1.084) for central nervous system tumors.

Regarding the claimed increasing hazard ratios for childhood cancer for each mSv increase in cumulative dose of external radiation, this would be expected to be the case irrespective of the cause of childhood cancer. This is because childhood cancer cumulative incidence increases with follow-up time, which is positively correlated with cumulative radiation dose from birth. Had cumulative exposure to air pollution, cumulative food intake, or cumulative water intake been used as the risk factor, a similar outcome would be expected because all are positively correlated with follow-up time.

The authors used in their analyses the doubly weighted (via radiation and tissue weighting factors) hypothetical effective dose rate and effective dose related to total-body irradiation from sources outside the body. They were apparently unaware that tissue weighting factors used are based on detriment rather than solely on cancer and that all significant contributions to radiation absorbed dose need to be accounted for. The authors omitted the very important contributions to radiation dose from radionuclides inside the body and from medical procedures. For example, the internal radiation dose to active bone marrow from radon and thoron can be significant (Richardson et al. 1991). Thus, misclassification of individuals to effective dose and dose rate groups likely occurred more frequently than acknowledged by Spycher et al. In addition, when focusing on a specific type of potential outcome (e.g., leukemia) and its association with radiation exposure of a specific target tissue (e.g., active bone marrow), it is better to use the equivalent dose, which involves only the radiation weighting factor, when a mixture of different radiations are involved (National Research Council 2006). This guards against unnecessary systematic error associated with using both the subjective radiation and tissue weighting factors to get the effective dose that was used by the authors.

The individual-specific radiation doses and dose rates assigned by Spycher et al. likely involved significant errors (statistical and systematic), with the dose error possibly being larger than the effective dose assigned to the individual. Part of the systematic error relates to neglected doses from other sources (e.g., internal radionuclides). Because the focus of the research was on very small radiation dose rates and small cumulative doses, it is important to address dose and dose rate errors when conducting such analyses. It is also important to address uncertainty associated with other confounding factors studied (traffic-related air pollution, electromagnetic fields from radio and TV transmitters and from high-voltage power lines, degree of urbanization of municipality, socioeconomic status, etc.) as well as errors related to the use of probabilistic record linkage between the Swiss Childhood Cancer Registry and the Swiss National Cohort. If such errors and uncertainty had been addressed in the authors’ logistic regression analyses, then it is likely that no association between background radiation and childhood leukemia would have been suggested by the research results obtained.
